# Factors Influencing Utilization of the Primary Prevention Implantable Defibrillator

**DOI:** 10.1371/journal.pone.0121515

**Published:** 2015-03-20

**Authors:** Lin Zhang, Kumar Narayanan, Harpriya Chugh, Takahiro Shiota, Zhi-Jie Zheng, Sumeet S Chugh

**Affiliations:** 1 Shanghai Jiaotong University School of Public Health, Shanghai, China; 2 The Heart Institute, Cedars-Sinai Medical Center, Los Angeles, California, United States of America; University of Louisville, UNITED STATES

## Abstract

**Background:**

A growing literature suggests underutilization of the primary prevention implantable cardioverter-defibrillator (ICD); thus, factors influencing utilization need to be understood. We performed a comprehensive assessment of patient characteristics and health insurance status among subjects eligible for primary prevention ICD in a tertiary care center.

**Methods:**

From among a group of patients who met criteria for primary prevention ICD based on left ventricular dysfunction (LVEF ≤ 35%), ICD recipients (n = 110) were compared to ICD non-recipients (n = 110) to identify determinants of ICD implantation. We evaluated demographics, clinical profile including Charlson Comorbidity Index [CCI, categorized as low (≤3) or high (>3)] and health insurance status.

**Results:**

ICD recipients were younger (62.1±15.0 vs. 68.0±18.2; P = 0.01), with more males (80% vs. 65.5%; P = 0.01), higher NYHA class (II/III: 75.5% vs. 40.2%; P<0.001) and more likely to have supplemental private health insurance (61.8% vs. 46.4%; P = 0.02). CCI was not significantly different between the two groups (low CCI 61.8% vs. 62.7%; P = 0.89). In multivariable analysis, factors independently associated with ICD implantation were male sex (OR, 2.77, [1.31-5.85]; P = 0.01), age<75 (OR, 2.68, [1.30-5.50]; P = 0.01), private insurance (OR, 2.17, [1.08-4.36], P = 0.03) and NYHA Class II/III (OR, 5.91, [2.91-12.01]; P<0.001). Documentation of discussion about primary prevention ICD was absent in the majority (57.2%) of non-recipients.

**Conclusion:**

In a contemporary urban tertiary care setting, age, sex and heart failure symptom class were associated with ICD utilization, with socioeconomic/insurance status also potentially playing a role. These findings have implications for optimizing appropriate utilization of the prophylactic ICD and warrant follow-up in larger, more diverse populations.

## Introduction

At least 300,000 cases of sudden cardiac death (SCD) are reported every year in the United States accounting for more than half of all cardiovascular deaths[1]. Despite considerable efforts in the field of resuscitation, survival following sudden cardiac arrest remains low [2]; hence improved prevention continues to be a priority. The efficacy of the implantable cardioverter defibrillator (ICD) in reducing mortality related to SCD when used for primary prevention in subjects with severe left ventricular (LV) systolic dysfunction (Ejection Fraction, EF ≤35%) has been well established [3, 4]. However, several reports suggest under-utilization of the primary prevention ICD in real world practice. A recent analysis from the Oregon Sudden Unexpected Death Study (Oregon SUDS) showed that among subjects who were guideline-eligible and eventually experienced SCD in the community, only 13.1% were implanted before the SCD event [5]. Even in heart failure populations, registry-based [6] as well as hospital-based [7] data have shown utilization rates ranging from 30–40%.

Factors determining ICD implantation for primary prevention in real world practice are likely to be varied and complex. Previous studies have suggested that different factors may play a role, including age [8], sex [9], race [10], comorbid conditions, physician and hospital related factors [8]. However, patterns of ICD utilization and the influencing factors may differ based on different patient sub-groups as well as health care settings. Optimal utilization of this important prevention modality is likely to require a detailed understanding of these issues. While studies have looked at different individual factors, it is likely that consideration of all potentially relevant factors in a common context would also be helpful. We therefore performed a comprehensive assessment of factors influencing primary prevention ICD deployment in a contemporary setting, at a U.S. urban tertiary care center.

## Methods

Consecutive adult patients with severe LV dysfunction (LVEF ⩽ 35%) were identified from the echocardiography laboratory database at Cedars-Sinai Medical Center, Los Angeles between February and October 2013. Detailed demographic, clinical and health insurance data as well as information on whether an ICD had been implanted for primary prevention were obtained from the electronic medical records system for all patients. Eligibility for primary prevention ICD implantation was assessed based on current guidelines [11, 12]. Patients who had ICD implantation for secondary prevention, those with EF assessments within 40 days of an acute coronary syndrome, subjects with newly diagnosed heart failure (< 3 months), those who lacked follow-up information and those with otherwise incomplete data were excluded from the study. Further, patients with prior heart transplantation, or mechanical circulatory support such as total artificial heart or left ventricular assist device were also excluded.

ICD implantations included single or dual chamber devices as well as cardiac resynchronization therapy with defibrillator (CRT-D). For the patients who were eligible but did not receive an ICD, we looked for documentation of physician discussion regarding primary prevention ICD as well as any evidence of patient refusal to undergo implantation. In order to determine factors associated with ICD implantation, primary prevention ICD recipients and non-recipients (those eligible but not implanted) were compared with respect to several clinical and demographic variables as well as health insurance status. Individuals with only Medicare/Medicaid/ Medi-Cal were classified as having ‘public insurance’. Individuals <65 years old with private health insurance (self or employer paid) and individuals ≥ 65 years old with supplemental private insurance in addition to Medicare/ Medicaid/ Medi-Cal were classified as having ‘private insurance’. Subjects without any form of insurance coverage were classified as having no insurance. The Charlson Comorbidity Index (CCI) was calculated for each patient in order to compare burden of comorbidity [13] and was categorized into low (CCI score ≤3) and high (CCI score > 3). For ICD recipients, parameters prior and closest to the ICD implantation were used.

### Statistical analysis

Statistical analysis was performed using SPSS 20 (IBM Corporation, NY). Continuous and categorical variables were expressed as mean ±SD and number (percentage) respectively. Variables between ICD recipients and non-recipients were compared using the ***χ*** ^***2***^ test or Fisher's exact test for categorical variables and independent samples ***t*** test for continuous variables. Multivariable logistic regression, incorporating all variables significant in univariate analysis, was used to identify factors independently associated with ICD implantation. A two-tailed P value of ≤0.05 was considered statistically significant. The study was approved by the Institutional Review Board of Cedars-Sinai Medical Center (IRB approval number: Pro00034077).

## Results

**Fig 1:**
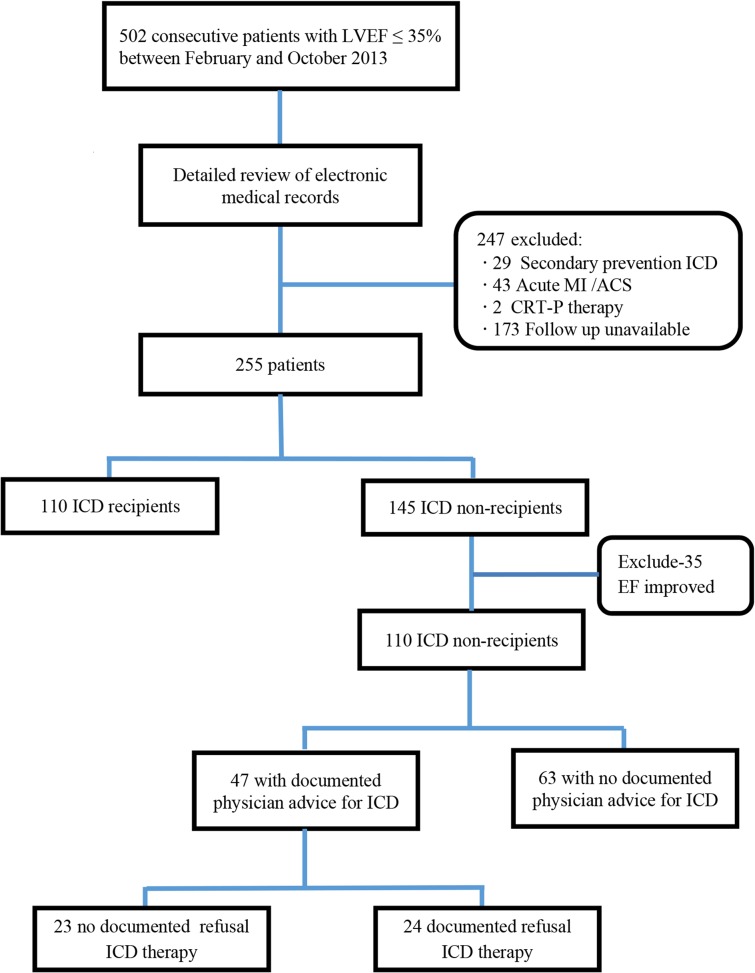
Flow Chart Indicating the Process of Identifying ICD Recipients and Non-Recipients.

A total of 502 patients with LVEF ≤ 35% were identified over a 9-month period. Adequate information was available for 255 patients who were guideline-eligible for a primary prevention ICD. Out of these 255 patients, 110 (43.1%) had a primary prevention ICD implanted (Fig. 1.).

After excluding 35 ICD non-recipients who had improvement of EF with medical therapy, 110 ICD recipients and 110 ICD non-recipients were compared to identify differences. Among the 110 ICD recipients, 69 (62.7%) were implanted with single or dual chamber devices, while 41 (37.3%) had CRTD implantation. Among the ICD non-recipients, 63 patients (57.2%) had no documentation of physician discussion regarding ICD implantation. Out of those patients with ICD implantation advised, 24 patients (51.1%) refused to undergo the procedure. A clear reason for refusal was not available in most cases; two patients were willing to accept risk of SCD, two patients had insurance issues, and one patient was awaiting re-assessment of EF after cessation of chronic alcohol use.

**Fig 2:**
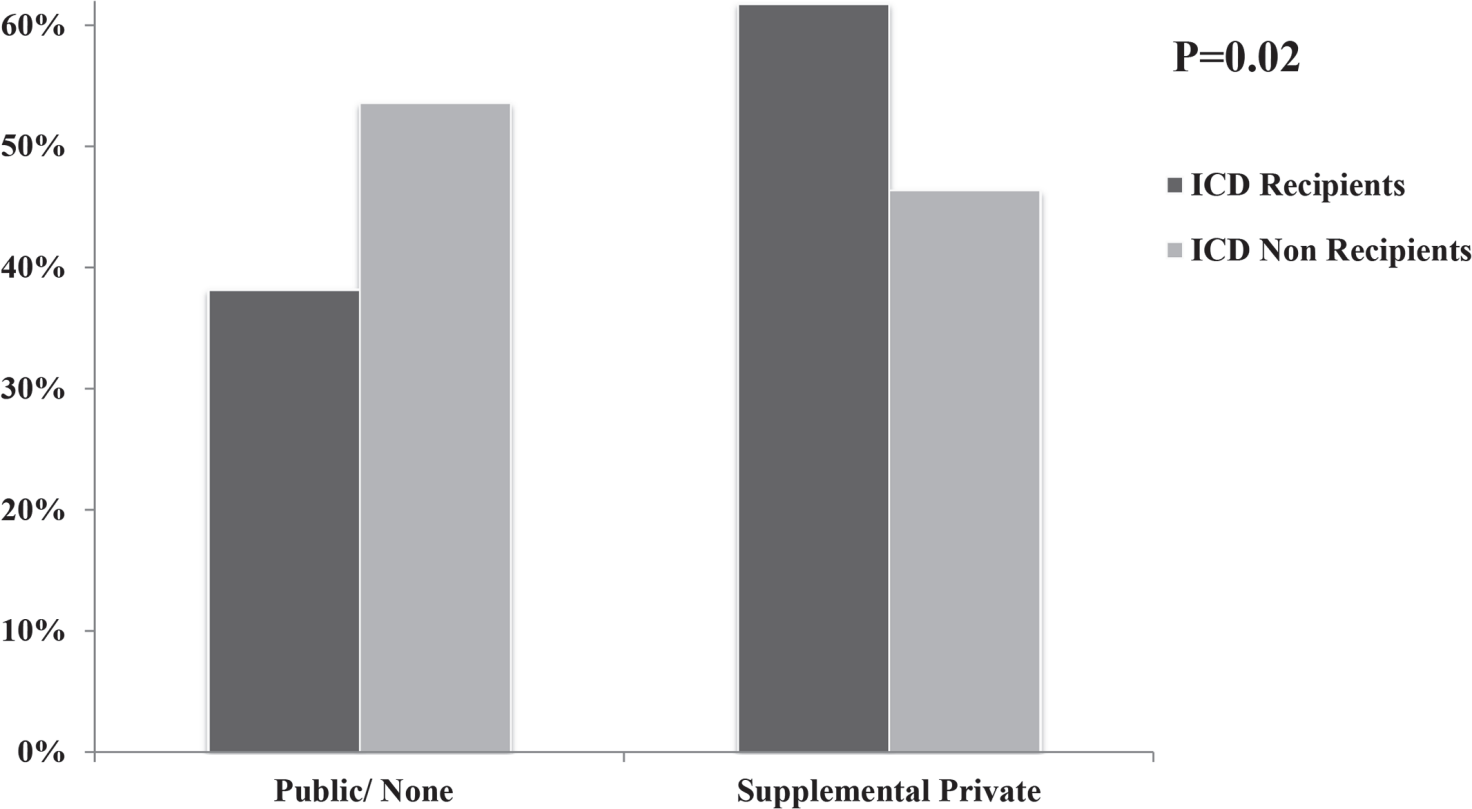
Health Insurance Status of ICD Recipients versus Non-Recipients.

The demographic characteristics of ICD recipients versus non-recipients are presented in Table 1. Compared to ICD non-recipients, ICD recipients were younger (62.1±15.0 vs. 68.0±18.2; P = 0.01) with greater proportion of males (80% vs. 65.5%; P = 0.01). Recipients were less likely to be current alcohol users (5.5% vs. 8.2%; P = 0.05), smokers (5.5% vs. 13.6%; P = 0.03) or have a history of drug abuse (0.9% vs. 10%; P<0.01). In addition, ICD recipients were more likely to have private (including combined public and supplemental private) health insurance (61.8% vs. 46.4%, P = 0.02) (Fig. 2.).There were no significant differences with respect to race between the two groups.

**Table 1:** Demographic Characteristics of ICD Recipients versus Non-Recipients.

	ICD recipients (N = 110)	ICD Non-recipients (N = 110)	P value
**Age**	62.1±15.0	68.0±18.2	0.01
**Age category**			0.01
** Under 75**	84(76.4)	65(59.1)	
** 75 and older**	26(23.6)	45(40.9)	
**Male**	88(80)	72(65.5)	0.01
**Obese** ^a^	33(30.6)	21(19.8)	0.07
**Race**			0.38
** Caucasian**	66(60)	65(59.1)	
** Black / AA**	30(27.3)	25(22.7)	
** Hispanic**	8(7.3)	6(5.5)	
** Others**	6(5.4)	14(12.7)	
**Health insurance type**			0.02
**Only Public/ None**	43(38.2)	59(53.6)	
**Supplemental Private**	68(61.8)	51(46.4)	
**Current alcoholism**	6(5.5)	9(8.2)	0.05
**Current smoker**	6(5.5)	15(13.6)	0.03
**Current drug abuse**	1(0.9)	11(10)	< 0.01

Results presented as mean ± SD for continuous variables and n (%) for categorical variables.

^a^Obesity defined as body mass index ≥30 kg/m^2^, data was available for 108 ICD recipients and 106 non-recipients.

Table 2 and Table 3 show the cardiac parameters and comorbidities respectively among the ICD recipients and non-recipients. ICD recipients were more likely to have coronary artery disease (56.4% vs. 41.8%; P = 0.03), and higher New York Heart Association (NYHA) functional class (class II—III 75.5% vs. 40.2%; P<0.001). The prevalence of other co-morbidities including diabetes, hypertension, liver disease and chronic kidney disease were not significantly different between the two groups. However, the proportion of patients undergoing dialysis was significantly lower in the ICD recipients group (6.4% vs. 14.5%; p = 0.05). When categorized by the CCI, there was no significant difference between ICD recipients and non-recipients (low CCI 61.8% vs. 62.7%; P = 0.89).

**Table 2:** Cardiac Parameters of ICD Recipients versus Non-Recipients.

	ICD recipients (N = 110)	ICD Non-recipients (N = 110)	P value
**Left ventricular ejection fraction (EF)**			0.14
** EF 30–35%**	28(25.5)	38(34.5)	
** EF<30%**	82(74.5)	72(65.5)	
**QRS duration^a^**			0.38
** QRS<120**	56(54.4)	64(60.4)	
** QRS≥120**	47(45.6)	42(39.6)	
**Coronary artery disease**	62(56.4)	46(41.8)	0.03
**Atrial fibrillation**	23(20.9)	32(29.1)	0.16
**Coronary artery bypass grafting**	20(18.2)	16(14.5)	0.47
**Percutaneous coronary intervention**	13(11.8)	14(12.7)	0.84
**NYHA Class II/III**	74(75.5)	41(40.2)	<0.001
**Beta blocker**	80(72.7)	77(70)	0.65
**Use of ACEI/ ARB^b^**	69(62.7)	52(47.3)	0.02

Results presented as mean ± SD for continuous variables and n (%) for categorical variables.

^a^Data on QRS duration were available for 103 ICD recipients and 106 ICD non-recipients.

^b^ACEI/ARB- Angiotensin converting enzyme inhibitor/Angiotensin receptor blocker.

**Table 3:** Comorbidity Data of ICD Recipients versus Non-Recipients.

	ICD recipients (N = 110)	ICD Non-recipients (N = 110)	P value
**Peripheral vascular disease**	11(10)	17(15.5)	0.22
**Cerebrovascular disease**	15(13.6)	14(12.7)	0.84
**Chronic obstructive pulmonary disease**	17(15.5)	7(6.4)	0.03
**Diabetes mellitus**	43(39.1)	45(40.9)	0.78
**Chronic kidney disease**	39(35.5)	34(30.9)	0.47
**Dialysis**	7(6.4)	16(14.5)	0.05
**Solid Tumor/Leukemia**	15(13.6)	12(10.9)	0.54
**Liver disease**	3(2.7)	5(5.5)	0.31
**HIV infection^a^**	2(1.8)	4(3.6)	0.41
**Charlson Comorbidity Index (CCI)**			0.89
** CCI ≤3**	68(61.8)	69(62.7)	
** CCI >3**	42(38.2)	41(37.3)	

^a^HIV- Human immunodeficiency virus.

In multivariable analysis, parameters independently associated with ICD recipient status included age <75 (OR 2.68, 95%CI 1.30–5.50; P = 0.01), male sex (OR 2.77, 95% CI 1.31–5.85; P = 0.01), private health insurance (OR 2.17, 95% CI 1.08–4.36; P = 0.03), and NYHA Class II/III (OR 5.91, 95% CI 2.91–12.01; P<0.001) (Table 4).

**Table 4:** Factors Independently Associated with ICD Utilization.

	Odds Ratio (95% C.I.)	P Value
**Age <75 vs≥75yrs**	2.68 (1.30–5.50)	0.01
**Male vs. Female**	2.77(1.31–5.85)	0.01
**Supplemental private vs. Public/No insurance**	2.17(1.08–4.36)	0.03
**Current alcoholism**	0.61(0.13–2.84)	0.53
**Current drug abuse**	0.12(0.01–1.24)	0.08
**Current smoker**	0.35(0.09–1.40)	0.14
**Use of ACEI/ARB**	1.89(0.95–3.73)	0.07
**Low CCI vs. High CCI^a^**	1.22(0.60–2.49)	0.57
**Coronary artery disease**	1.86(0.93–3.74)	0.08
**NYHA Class II/III vs. NYHA Class I**	5.91(2.91–12.01)	<0.001

95% CI = 95% confidence interval.

^a^CCI score was categorized as low (CCI score ≤3) and high (CCI score >3); CCI-Charlson comorbidity index.

## Discussion

We performed a comprehensive evaluation of the factors influencing primary prevention ICD utilization in a real-world clinical setting by simultaneously considering a combination of clinical, demographic and health insurance factors. We identified significant differences between ICD recipients and non-recipients among patients who were guideline-eligible. In multivariable analysis, age<75 years, male sex, NYHA symptom class and having private health insurance remained independently associated with ICD recipient status. The results of this study potentially reveal important determinants of ICD utilization in actual clinical practice and help identify areas for improvement in optimizing appropriate use of the ICD for primary prevention. While an implantation rate of 43% appears to be lower than expected, this may be partly influenced by referral bias, with sicker, more challenging patients being selectively referred for quaternary care.

We found that in >50% of ICD non-recipients, there was no documentation of physician discussion about primary prevention ICD implantation. While it is difficult to draw firm conclusions, prior studies have reported that there may be lack of awareness of guidelines even among specialists [14]. Further, differences in individual physician perceptions may influence decision-making process [15, 16]. Targeted health care provider education regarding primary prevention guidelines may have a role in improving ICD utilization rates and needs further study.

The factors found to be associated with ICD utilization in our study are in agreement with previous work in this field. Age is uniformly seen to be an important factor, with older candidates less likely to undergo implantation [5, 8], which may reflect physician judgment as well as potentially greater likelihood of refusal among older individuals. Consistent with earlier reports, we also observed that men were more likely to undergo implantation [10, 17]. It has been suggested that sex differences in implantation rates are not due to relative over-utilization of the ICD in men, but rather reflects true under-utilization in women [18]. We did not find a significant difference in the comorbidity burden between ICD recipients and non-recipients in our study, although previous studies show that patients with greater comorbidity are less likely to get an ICD [8, 19]. Most ICD trials excluded subjects with significant comorbidity and specific studies evaluating the benefit of the primary prevention ICD in such populations are urgently needed.

Our finding that patients with private health insurance were more likely to get an ICD needs careful consideration to understand its potential implications. We did not find evidence to suggest that any patient had been denied ICD implantation based on insurance status and such an interpretation may be somewhat simplistic and potentially erroneous. Nevertheless, other studies have also found a link between insurance status and implantation rates [20]. It is likely that the health insurance status is a broader reflector of socio-economic status, which could influence healthcare access and utilization in diverse ways. For instance, type of health insurance was found to correlate with type of employment as well as overall income and assets and predicted decline in physical function over time [21]. ICD therapy is not only expensive, but also requires sustained efforts on the part of the patient and family to seek ongoing care over an extended period of time, which can be challenging for the economically underprivileged [22].

Other studies have reported that the primary prevention ICD may be potentially under used [5–7]. However, data from the National Cardiovascular Data Registry (NCDR) ICD registry indicate that 22.5% of the patients had non-evidence based implants [23], suggesting possible over implantation. While this may seem paradoxical, these two issues are likely to represent two different facets of the problem, i.e., on the one hand a proportion of eligible candidates do not get the device and on the other, some are being implanted inappropriately. The key issue therefore is not whether more or fewer ICDs need to be implanted but in rather ensuring that ICDs are implanted in the candidates *most likely to benefit*. What measures can we take towards this goal? Referral for primary prevention ICD should be guideline-directed, but also consistent with appropriate clinical care. When designing strategies to maximize such appropriate referral it may be important for institutions to incorporate demographic and clinical factors most pertinent to local practice. Assessment of existing implantation rates and identification of potential roadblocks through studies such as the present one is likely to be a helpful tool. Measures such as computerized decision support tools, automated referral to heart rhythm services if EF ≤ 35%, regular internal audits and other quality improvement processes contribute to improving appropriate implantation rates. In a single center study, Gupta et al. showed that a simple reminder note to providers in the electronic medical system improved both rates of discussion about ICD with the patient as well as actual referral for implantation [24]. Additionally, the efforts of initiatives such as the NCDR ICD database that collects metrics for primary prevention ICD implantations is likely to also contribute to improving ICD utilization [25]. Lastly, the low specificity of EF as a risk predictor, with resultant high number needed to treat [4, 26] suggests the need for ongoing research to identify better risk stratification approaches for primary prevention.

## Limitations

One of the strengths of the present analysis is that it was performed through detailed review of individual patient records, which is likely to be more accurate than use of International Classification of Diseases diagnostic codes [27]. However, we acknowledge some limitations. This was a single center study with some attendant bias. Though it would have been useful to have county-level or NCDR data for comparison, this was beyond the scope of the present study and could form the basis for future investigation. Clinical practice patterns may vary across hospitals and our results may not be generalizable to other settings [28]. As in any observational study, unmeasured confounders may influence results. However, in identifying some important factors associated with ICD utilization in contemporary practice, our study can serve as the first step for more in-depth research in this area. Finally, our study design did not allow for evaluation of follow-up information on outcomes including mortality in the ICD recipients and non-recipients.

## Conclusions

Age, sex and heart failure symptom class were associated with primary prevention ICD utilization in a real world clinical practice setting. In addition, socio-economic factors and insurance status may also play a role. This important area needs further investigation in diverse communities and settings with the goal of optimizing appropriate use of the primary prevention ICD in the community.

## References

[1] Myerburg RJ, Junttila MJ. Sudden cardiac death caused by coronary heart disease. Circulation. 2012;125(8):1043–52. 10.1161/CIRCULATIONAHA.111.023846 22371442

[2] Nichol G, Thomas E, Callaway CW, Hedges J, Powell JL, Aufderheide TP, et al Regional variation in out-of-hospital cardiac arrest incidence and outcome. Jama. 2008;300(12):1423–31. 10.1001/jama.300.12.1423 18812533 PMC3187919

[3] Moss AJ, Zareba W, Hall WJ, Klein H, Wilber DJ, Cannom DS, et al Prophylactic implantation of a defibrillator in patients with myocardial infarction and reduced ejection fraction. N Engl J Med. 2002;346(12):877–83. 11907286 10.1056/NEJMoa013474

[4] Bardy GH, Lee KL, Mark DB, Poole JE, Packer DL, Boineau R, et al Amiodarone or an implantable cardioverter-defibrillator for congestive heart failure. N Engl J Med. 2005;352(3):225–37. 15659722 10.1056/NEJMoa043399

[5] Narayanan K, Reinier K, Uy-Evanado A, Teodorescu C, Chugh H, Marijon E, et al Frequency and determinants of implantable cardioverter defibrillator deployment among primary prevention candidates with subsequent sudden cardiac arrest in the community. Circulation. 2013;128(16):1733–8. 10.1161/CIRCULATIONAHA.113.002539 24048201

[6] Hernandez AF, Fonarow GC, Liang L, Al-Khatib SM, Curtis LH, LaBresh KA, et al Sex and racial differences in the use of implantable cardioverter-defibrillators among patients hospitalized with heart failure. JAMA. 2007;298(13):1525–32. 17911497 10.1001/jama.298.13.1525

[7] Bradfield J, Warner A, Bersohn MM. Low referral rate for prophylactic implantation of cardioverter-defibrillators in a tertiary care medical center. Pacing and clinical electrophysiology: PACE. 2009;32 Suppl 1:S194–7. 10.1111/j.1540-8159.2008.02281.x 19250092

[8] Chae SH, Koelling TM. Patient and physician determinants of implantable cardioverter defibrillator use in the heart failure population. Congestive heart failure. 2010;16(4):141–6. 10.1111/j.1751-7133.2009.00139.x 20662865

[9] Curtis LH, Al-Khatib SM, Shea AM, Hammill BG, Hernandez AF, Schulman KA. Sex differences in the use of implantable cardioverter-defibrillators for primary and secondary prevention of sudden cardiac death. JAMA. 2007;298(13):1517–24. 17911496 10.1001/jama.298.13.1517

[10] Mezu U, Ch I, Halder I, London B, Saba S. Women and minorities are less likely to receive an implantable cardioverter defibrillator for primary prevention of sudden cardiac death. Europace. 2012;14(3):341–4. 10.1093/europace/eur360 22071382

[11] Epstein AE, Dimarco JP, Ellenbogen KA, Estes NA 3rd, Freedman RA, Gettes LS, et al ACC/AHA/HRS 2008 Guidelines for device-based therapy of cardiac rhythm abnormalities. Heart Rhythm. 2008;5(6):e1–62. Epub 2008/06/07. 10.1016/j.hrthm.2008.04.014 18534360

[12] Tracy CM, Epstein AE, Darbar D, DiMarco JP, Dunbar SB, Estes NAM, et al 2012 ACCF/AHA/HRS Focused Update of the 2008 Guidelines for Device-Based Therapy of Cardiac Rhythm AbnormalitiesA Report of the American College of Cardiology Foundation/American Heart Association Task Force on Practice Guidelines. Journal of the American College of Cardiology. 2012;60(14):1297–313. 10.1016/j.jacc.2012.07.009 22975230

[13] Charlson ME, Pompei P, Ales KL, MacKenzie CR. A new method of classifying prognostic comorbidity in longitudinal studies: development and validation. Journal of chronic diseases. 1987;40(5):373–83. 3558716 10.1016/0021-9681(87)90171-8

[14] Hubinette C, Lund LH, Gadler F, Stahlberg M. Awareness of indications for device therapy among a broad range of physicians: a survey study. Europace: European pacing, arrhythmias, and cardiac electrophysiology: journal of the working groups on cardiac pacing, arrhythmias, and cardiac cellular electrophysiology of the European Society of Cardiology. 2014;16(11):1580–6. 10.1093/europace/eut416 24451291

[15] McHale B, Harding SA, Lever NA, Larsen PD. A national survey of clinician's knowledge of and attitudes towards implantable cardioverter defibrillators. Europace. 2009;11(10):1313–6. 10.1093/europace/eup236 19734156

[16] Castellanos JM, Smith LM, Varosy PD, Dehlendorf C, Marcus GM. Referring physicians' discordance with the primary prevention implantable cardioverter-defibrillator guidelines: A national survey. Heart Rhythm. 2012;9(6):874–81. 10.1016/j.hrthm.2012.01.029 22306794 PMC3355218

[17] Al-Khatib SM, Hellkamp AS, Hernandez AF, Fonarow GC, Thomas KL, Al-Khalidi HR, et al Trends in use of implantable cardioverter-defibrillator therapy among patients hospitalized for heart failure: have the previously observed sex and racial disparities changed over time? Circulation. 2012;125(9):1094–101. 10.1161/CIRCULATIONAHA.111.066605 22287589 PMC3670671

[18] Daugherty SL, Peterson PN, Wang Y, Curtis JP, Heidenreich PA, Lindenfeld J, et al Use of implantable cardioverter defibrillators for primary prevention in the community: do women and men equally meet trial enrollment criteria? Am Heart J. 2009;158(2):224–9. 10.1016/j.ahj.2009.05.018 19619698

[19] Saba S, Ravipati LP, Voigt A. Recent trends in utilization of implantable cardioverter-defibrillators in survivors of cardiac arrest in the United States. Pacing and clinical electrophysiology: PACE. 2009;32(11):1444–9. 10.1111/j.1540-8159.2009.02509.x 19712076

[20] LaPointe NM, Al-Khatib SM, Piccini JP, Atwater BD, Honeycutt E, Thomas K, et al Extent of and reasons for nonuse of implantable cardioverter defibrillator devices in clinical practice among eligible patients with left ventricular systolic dysfunction. Circulation Cardiovascular quality and outcomes. 2011;4(2):146–51. 10.1161/CIRCOUTCOMES.110.958603 21304098

[21] Kim J, Richardson V. The impact of socioeconomic inequalities and lack of health insurance on physical functioning among middle-aged and older adults in the United States. Health & social care in the community. 2012;20(1):42–51. 21733029 10.1111/j.1365-2524.2011.01012.x

[22] Udell JA, Juurlink DN, Kopp A, Lee DS, Tu JV, Mamdani MM. Inequitable distribution of implantable cardioverter defibrillators in Ontario. International journal of technology assessment in health care. 2007;23(3):354–61. 17579939 10.1017/s0266462307070389

[23] Al-Khatib SM, Hellkamp A, Curtis J, Mark D, Peterson E, Sanders GD, et al Non-evidence-based ICD implantations in the United States. JAMA. 2011;305(1):43–9. 10.1001/jama.2010.1915 21205965 PMC3432303

[24] Gupta A, Gholami P, Turakhia MP, Friday K, Heidenreich PA. Clinical reminders to providers of patients with reduced left ventricular ejection fraction increase defibrillator referral: a randomized trial. Circulation Heart failure. 2014;7(1):140–5. 10.1161/CIRCHEARTFAILURE.113.000753 24319096

[25] Hammill SC, Kremers MS, Stevenson LW, Heidenreich PA, Lang CM, Curtis JP, et al Review of the registry's fourth year, incorporating lead data and pediatric ICD procedures, and use as a national performance measure. Heart Rhythm. 2010;7(9):1340–5. 10.1016/j.hrthm.2010.07.015 20647056

[26] Moss AJ, Greenberg H, Case RB, Zareba W, Hall WJ, Brown MW, et al Long-term clinical course of patients after termination of ventricular tachyarrhythmia by an implanted defibrillator. Circulation. 2004;110(25):3760–5. 15583079 10.1161/01.CIR.0000150390.04704.B7

[27] Hoang A, Shen C, Zheng J, Taylor S, Groh WJ, Rosenman M, et al Utilization rates of implantable cardioverter-defibrillators for primary prevention of sudden cardiac death: a 2012 calculation for a midwestern health referral region. Heart Rhythm. 2014;11(5):849–55. 10.1016/j.hrthm.2014.02.019 24566233 PMC4074531

[28] Shah B, Hernandez AF, Liang L, Al-Khatib SM, Yancy CW, Fonarow GC, et al Hospital Variation and Characteristics of Implantable Cardioverter-Defibrillator Use in Patients With Heart FailureData From the GWTG-HF (Get With The Guidelines–Heart Failure) Registry. Journal of the American College of Cardiology. 2009;53(5):416–22. 10.1016/j.jacc.2008.09.045 19179199

